# Mood States, Injury Status, and Countermovement Jump Performance in Brazilian High-Level Sports

**DOI:** 10.3390/sports13090303

**Published:** 2025-09-03

**Authors:** Izabel Cristina Provenza de Miranda Rohlfs, Franco Noce, Carolina F. Wilke, Tim J. Gabbett, Victoria R. Terry, Alexandre Montenegro, Carlos Alexandre Assis, Paula Moreira Magalhães, Pasteur O. de Miranda, Peter C. Terry

**Affiliations:** 1 School of Psychology and Wellbeing, University of Southern Queensland, Toowoomba 4350, Australia; peter.terry@unisq.edu.au; 2 Unified Center for the Identification and Development of Performance Athletes (CUIDAR), Clube de Regatas do Flamengo, Rio de Janeiro 22430-041, Brazil; alexandre.montenegro@flamengo.com.br (A.M.); carlos.assis@flamengo.com.br (C.A.A.); paula.magalhaes@flamengo.com.br (P.M.M.); 3 School of Physical Education, Physiotherapy and Occupational Therapy, Federal University of Minas Gerais, Belo Horizonte 31270-901, Brazil; fnoce@hotmail.com (F.N.); carolina.wilke@stmarys.ac.uk (C.F.W.); 4 Faculty of Sport, Allied Health, and Performance Sciences, St. Mary’s University, Twickenham, London TW1 4SX, UK; 5 Gabbett Performance Solutions, Brisbane 4011, Australia; tim@gabbettperformance.com.au; 6 School of Nursing and Midwifery, University of Southern Queensland, Toowoomba 4350, Australia; victoria.terry@unisq.edu.au; 7 Independent Researcher, Belo Horizonte 30315-030, Brazil; pasteurjr@gmail.com; 8 Centre for Health Research, University of Southern Queensland, Toowoomba 4350, Australia

**Keywords:** injury prevention, neuromuscular performance, psychological well-being, athlete monitoring, Brazil Mood Scale (BRAMS)

## Abstract

This study investigated relationships between mood profiles, sports injuries, and countermovement jump with arm swing (CMJ) performance in a cohort study of 417 Brazilian athletes using a multi-methods approach during the period from January to November 2023. Six distinct mood profiles were identified, termed the shark fin (28.3%), iceberg (20.4%), submerged (18.7%), inverse iceberg (18.0%), surface (9.8%), and inverse Everest (4.8%). Athletes with the inverse Everest profile had a significantly higher risk of injury (OR = 2.90; 90% CI [1.09–7.55]) compared to those with the iceberg profile. Random forest models showed moderate predictive capability (AUC = 0.651), with vigour (12.7%) and anger (11.5%) as primary predictors. Bayesian analysis confirmed a higher injury probability in athletes with the inverse Everest profile (31.8%). Despite statistical power limitations, the results indicate that negative mood profiles, particularly those with elevated anger and fatigue, are associated with increased injury risk. Mood scores were not associated with CMJ performance variation. These findings highlight the importance of considering mood profiles in athlete monitoring systems, acknowledging the complex interplay between psychological and physical factors in injury risk.

## 1. Introduction

Mood profiling, a process in which an individual’s mood scores are plotted against normative data to create a graphical profile [1], is used to identify patterns of mood responses. This procedure is often applied in research exploring links between mood, performance, and psychological well-being in athletes and exercisers. Conceptually, moods are low-intensity feeling states that, unlike emotions, tend to lack specific triggers. Less intense but longer lasting than emotions, moods vary in valence (positive to negative) and arousal (activation to deactivation) [2,3,4].

The Brunel Mood Scale (BRUMS) [5] has been applied across a variety of sporting and cultural contexts to assess mood states and their implications for performance [6], well-being [7], and injury risk [8]. The BRUMS measures six dimensions of mood—tension, depression, anger, vigour, fatigue, and confusion—providing a snapshot of an athlete’s emotional state. Derived from the Profile of Mood States (POMS) [9], the BRUMS was designed as a streamlined, context-specific tool for use in sport and exercise settings. With 24 items, the BRUMS offers a practical and time-efficient alternative to the original 65-item POMS, facilitating its application in settings where regular and/or brief psychological monitoring is valuable, such as athletic training and competitive environments. Beedie et al. [10] suggested that recognising and managing moods and emotions is beneficial for sustainable mental health, optimal performance, and quality of life.

The BRUMS has been adapted for use across at least 16 linguistic and cultural contexts, including Brazil [11,12,13,14,15,16,17,18]. In Brazil, the Brunel Mood Scale was validated as the Brazil Mood Scale (BRAMS) [18], which has since demonstrated strong psychometric properties and widespread applicability in sport contexts. The BRAMS was recently reassessed for its psychometric integrity, supporting use of both a retrospective response timeframe (“how have you been feeling over the past week, including today”) and an immediate response timeframe (“how are you feeling right now”) [19]. Six specific mood profile clusters are identified in the literature, referred to as the iceberg, inverse iceberg, inverse Everest, shark fin, submerged, and surface profiles [20] which were all clearly identified in a sample of Brazilian athletes using both response timeframes [21].

Andrade et al. [22,23] first linked negative mood states—particularly tension, depression, and fatigue—to injury and impaired performance in elite volleyball athletes. Brandt et al. [24] confirmed the performance benefits of the iceberg profile in elite sailors, while Goulart et al. [25] showed that delayed mood recovery was associated with performance decline in female footballers. Additional studies by Brandt et al. [26], da Silva et al. [27], and da Silva Moura et al. [28] extended these findings to combat sports, beach volleyball, and adaptive sports, respectively, underscoring associations between BRAMS, performance, and injury risk.

Although mood profiling has emerged as a salient method in athlete monitoring—providing psychological insights relevant to injury prediction and well-being—it appears to hold limited predictive utility for neuromuscular performance measures. In this context, jumping is a basic motor skill central to many sports, including basketball, gymnastics, and volleyball. Given the obvious relationship between jumping ability (i.e., muscular power) and success in sports competitions, it is advantageous to assess athletes’ ability to generate power with the lower limbs. Considering its importance and relative ease of measurement, we used countermovement jump (CMJ) testing as an indicator of lower-limb power and neuromuscular readiness. However, evidence suggests that mood states, including transient emotional conditions such as fatigue or tension, do not substantially correlate with CMJ outcomes. For instance, Feria-Madueño et al. [29] found no significant association between POMS scores and CMJ performance among elite Cuban athletes. Similarly, prior research underscores that CMJ height and kinetics are influenced by physiological determinants such as eccentric rate of force development, peak concentric power, and jump impulse, rather than psychological variables [30,31]. These findings suggest a dissociation between affective states and neuromuscular performance outputs, challenging assumptions that mood fluctuations translate into changes in explosive athletic ability. Hence, mood assessments may be more appropriately positioned as indicators of psychophysiological strain or readiness for cognitive and emotional regulation, rather than as proxies for motor performance.

Mood profiling has emerged as a crucial area of study in high-performance sport, offering significant insights into the complex interplay between an athlete’s psychological state, competitive performance, and susceptibility to injury. The literature consistently highlights the bidirectional relationship between an athlete’s mental well-being and their physical outcomes. For instance, a study by Galambos et al. [8] demonstrated that specific mood dimensions—notably vigour, depression, and tension—could account for a substantial portion (50%) of the variance in injury prevalence among elite athletes. This evidence suggests that mood states are powerful indicators of both performance potential and injury risk.

Longitudinal assessments of an athlete’s mood provide a valuable tool for understanding their psychological readiness and potential for injury. The iceberg profile, characterised by high vigour and low negative moods, is associated with successful performance and reduced injury risk across various sports [6,8,32]. This profile underscores the role of psychological resilience in buffering athletes against the stressors inherent in competition. Conversely, a mood profile marked by heightened negative emotional states is strongly associated with a decline in performance and increased injury susceptibility [33]. Psychological stress, often exacerbated by negative moods, can compromise an athlete’s immune system, impair cognitive function, and heighten their vulnerability to physical injury [34]. This makes mood monitoring a vital component of an overall health strategy, as it can provide early indications of stress overload, enabling timely intervention.

Contemporary research has moved away from a reductionist view of sports injuries to embrace a more holistic, systems-based model. The work of Bittencourt et al. [34] and Clement [35] posits that injuries result from a dynamic interplay between physical, psychological, and environmental factors, rather than a single isolated cause. This perspective advocates for a comprehensive, athlete-centred monitoring system that accounts for the complex, non-linear interactions between variables such as training load, emotional regulation, and biomechanics. Within this framework, mood profiling instruments like the BRAMS facilitate regular tracking of affective states, providing early warning signals of psychological strain that enable practitioners to implement individualised interventions before issues escalate [34,35]. As Fullagar et al. [36] have emphasised, the effective translation of this interdisciplinary knowledge into actionable strategies for coaches and medical staff is critical for fostering a more proactive and anticipatory approach to injury prevention, thereby supporting the dual objectives of performance optimisation and long-term athlete well-being [7].

The first aim of this study was to investigate whether mood profiles would be associated with injury occurrence during three to four months of training among high-performance Brazilian athletes. It was hypothesised (H1) that mood profiles would be significantly linked to injury occurrence. The second aim was to examine the association between mood scores and CMJ performance improvement during the same period of training. Given the nature of explosive, short-duration tasks like CMJ, it was hypothesised (H2) that the relationship with mood would not be significant.

## 2. Materials and Methods

### 2.1. Participants

This was a cohort study that included 417 athletes (female = 188, male = 229) from a prominent sports club in Rio de Janeiro, Brazil. The inclusion criteria required having at least three years of experience in high-performance training but there were no specific exclusion criteria. All athletes were affiliated with the Unified Centre for the Identification and Development of Performance Athletes (CUIDAR), a comprehensive programme designed to optimise athletic performance and well-being. CUIDAR, meaning “to care” in Portuguese, includes a multidisciplinary team of health professionals who collaborate closely with coaching and administrative staff. This integrated framework aims to enhance athletic performance while prioritising the safeguarding of athletes’ physical and mental health. The programme currently supports the development and well-being of over 1000 athletes through a team of health professionals from psychology, strength and conditioning, massage therapy, nutrition, social service, medicine, nursing, physiotherapy, and sport science. Athletes came from eight sports (artistic swimming, basketball, gymnastics, judo, rowing, swimming, volleyball, and water polo) and were aged between 12 and 44 years (M = 17.72 ± 4.54 years). Details of the participants’ characteristics are shown in Table 1.

### 2.2. Measurement of Mood

Mood was assessed using the BRAMS [18,19], a psychometrically validated 24-item scale comprising six subscales: anger (raiva), confusion (confusão), depression (depressão), fatigue (fadiga), tension (tensão), and vigour (vigor). The BRAMS demonstrated satisfactory internal consistency in the present study, as indicated by Cronbach’s alpha coefficients: anger (α = 0.90), confusion (α = 0.79), depression (α = 0.87), fatigue (α = 0.85), tension (α = 0.75), and vigour (α = 0.78). Although BRAMS demonstrates adequate validity and internal consistency as a tool for assessing mood dimensions, it is important to note that it is not suitable for diagnosing clinical depression nor other psychological disorders [5].

Participants reported their feelings *over the past week including today*, rating each item on a 5-point Likert scale: 0 = not at all (nada), 1 = a little (um pouco), 2 = moderately (moderadamente) 3 = quite a bit (bastante), and 4 = extremely (extremamente). The resulting subscale scores ranged from 0 to 16. Retrospective assessments are particularly useful in sport training contexts [32,37] as they help to detect whether mood has been negatively influenced by intense training loads or other sport-related factors. Besides affording a more practical and objective monitoring approach, requiring only a single measurement at the end of a training microcycle, the “past week” response timeframe enables coaches and multidisciplinary support teams to devise targeted strategies for preventing injuries, overtraining, and mental health issues. Thus, the “past week” response timeframe was used in the present study.

### 2.3. Measurement of Countermovement Jump with Arm Swing (CMJ) Performance

Physical performance was assessed using the CMJ test. Research by Heishman et al. [38] suggested that adding an arm swing to the CMJ test can improve sport-specificity and test reliability, making it particularly valuable for evaluating performance changes over time. Gathercole and colleagues [39] proposed that the CMJ is effective in detecting neuromuscular fatigue, indicating its usefulness for both youth and adult athletes in tracking performance adaptations and readiness. Moreover, the CMJ test either with or without arm swing is a reliable indicator of lower limb power, enabling coaches to monitor performance improvements or declines over time and providing insightful evaluations of training programme effectiveness. By assessing CMJ performance prior to training sessions, coaches can ascertain an athlete’s readiness and, where appropriate, adjust the session to optimise performance, and mitigate injury risk [40,41].

### 2.4. Injury Classification and Control

Athletic injuries are classified into two primary categories: acute and overuse injuries. Acute injuries occur abruptly, typically resulting from trauma or a specific incident, such as a fall, collision, or improper movement. Common examples include sprains, fractures, dislocations, and muscle strains, which often cause immediate pain, swelling, and impaired function [42]. Conversely, overuse injuries emerge gradually due to repetitive stress or microtrauma, and are frequently associated with excessive training loads, poor technique, or insufficient recovery. Examples include tendinitis, bursitis, and stress fractures, which often begin as mild discomfort but can escalate if unaddressed [35]. Preventing and managing both injury types necessitates tailored strategies, including injury monitoring, optimising training techniques, and ensuring adequate recovery.

Within the CUIDAR framework, a comprehensive injury database is meticulously maintained within an electronic medical record. For the present study, injury data from April to July 2023 was collected. This database serves as a centralised repository for documenting and tracking athlete injuries, thereby facilitating informed decision-making and evidence-based practice. Each injury is documented in a standardised spreadsheet, ensuring consistency and accuracy in data collection. The dataset comprises a range of variables, including athlete name and age, sport discipline and competitive category, date of physiotherapy referral, injury description and classification (overuse or acute), anatomical location and affected tissue, extent of training restriction (partial or total), and attending physician. By capturing these variables, the CUIDAR injury database provides a rich source of information for the multidisciplinary team, coaches, and athletes. This data can be leveraged to inform injury prevention strategies, optimise treatment protocols, and enhance overall athlete health and performance.

### 2.5. Procedure

Baseline CMJ data using the JumpTest technology [JumpTest, Belo Horizonte, MG, Brazil] were collected during the period from January to March 2023 as part of a pre-season athlete assessment protocol to provide baseline health and physical condition data to identify key aspects for training. All strength and conditioning coaches who conducted the testing followed the CUIDAR Performance Assessment Protocol and were trained to use the calibrated CMJ jump mat, which had been validated against a force platform [43]. The JumpTest uses a contact platform measuring 60 cm wide and 100 cm in length, which is sensitive to small pressures. The JumpTest Pro software version 2.10 was used in the present study.

A mid-season assessment of all athletes was carried out between July to November 2023 in which the CMJ was used to compare the performance of the athletes at the beginning of the season using the same Performance Assessment Protocol. The protocol included a specific warm-up regimen (10 jumping jacks + 5 body weight squats in the CMJ position + 10 jumping jacks + 5 warm up jumps in the same position as CMJ), followed by a 1 min. rest period prior to the initiation of the jumping trials. A series of three jumping attempts was conducted, with a 15 s inter-trial interval, and the average performance across the three attempts was computed and used in the subsequent analysis. The JumpTest mat has demonstrated strong validity and practical utility as a field-based tool for assessing lower-limb explosive performance [43], comparable to laboratory instruments when deployed within rigorously defined protocols. This approach is particularly pertinent given that lower-extremity muscle strength is a critical determinant of lower-limb functional capacity, as evidenced in both athletic and clinical populations [44,45].

BRAMS data were collected between April and July 2023, with every athlete completing a digital questionnaire via Google Forms on one occasion each. Athletes completed the questionnaire on their mobile devices under the supervision of team staff, including coaches and strength and conditioning specialists, all of whom had been trained to ensure the accurate administration of the BRAMS. Data collection occurred in the athletes’ typical training environments to reflect their habitual psychological states. The assessment of mood specifically evaluated how athletes had been feeling over the past week including that day, with the mood assessment taking place at the end of each week, either before or after their final training session. This ensured that the data captured reflected the athletes’ mood responses to their regular training loads.

**Figure 1:**
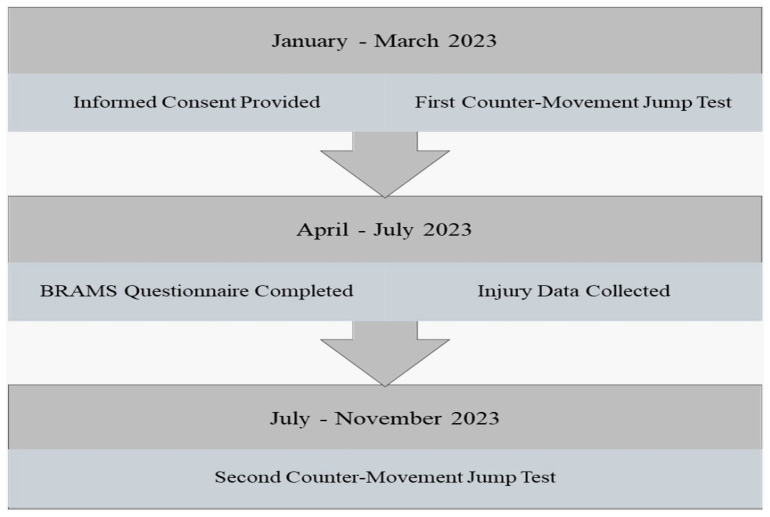
Data Collection Process and Timeline.

All participants were advised of their rights, and informed consent was obtained before data collection commenced. Athletes were made aware that their participation was voluntary and that they could withdraw from the study at any time without consequence. The research protocol received ethical approval from the University of Southern Queensland Human Research Ethics Committee (approval number: ETH2023-0046). This approach ensured that the collection process was both ethically sound and contextually relevant, providing a comprehensive snapshot of athletes’ mood states during a critical competitive training phase. A timeline of the research process is shown in Figure 1.

### 2.6. Data Analysis

A multi-method analytical strategy was employed to examine the associations among mood profiles, injury incidence, and performance outcomes. Data were processed using IBM SPSS Statistics version 29 (IBM Corp., Armonk, NY, USA) [46]. Mood scores were converted into T-scores based on normative Brazilian athlete data [18,19], enabling consistent classification into recognised profiles (e.g., iceberg, inverse Everest).

Exploratory analyses provided descriptive statistics and visualised variable distributions. Inferential analyses included MANOVA to compare mood scores between injured and uninjured athletes, as well as pre- and post-injury within the injured group. Discriminant function analysis (DFA) assessed classification accuracy for injury status, with attention to base rate, sensitivity, and specificity. Logistic regression with ElasticNet regularisation [47] modelled injury risk, addressing multicollinearity and improving predictive validity.

Survival analyses, including Kaplan–Meier curves and Cox models, evaluated the temporal relationship between mood and injury occurrence. Random forest models [48,49] ranked mood dimensions by predictive importance, while Bayesian estimation quantified injury probabilities across mood profiles. Bootstrap resampling (1000 iterations) yielded robust confidence intervals for odds ratios.

Chi-squared tests examined associations between injury type and sex. Further MANOVA and DFA compared mood profiles of athletes who improved versus those who did not improve in CMJ performance. Exact *p*-values and partial eta-squared (η^2^) were reported, with benchmarks of 0.01, 0.06, and 0.14 denoting small, medium, and large effect sizes, respectively [46]. Analyses accounted for sample size limitations, particularly in under-represented mood profiles (e.g., inverse Everest, *n* = 20) and the pre-/post-injury subgroup (*n* = 41).

## 3. Results

### 3.1. Data Screening

All participants completed the BRAMS “past week” assessment via an online questionnaire. As a result, there were no missing or out-of-range responses. Regarding univariate normality, the BRAMS subscales for anger, confusion, and depression exhibited non-normal distributions, which are commonly observed in negatively valenced mood dimensions. These dimensions tend to present a high proportion of low scores and fewer high scores [5,18,29,37]. Given the clinical and applied relevance of individuals exhibiting elevated scores on negative mood indicators—particularly in the context of mental health monitoring—such cases were retained in the dataset.

As previously recommended by Nevill and Lane [50], transformation of interval-level self-report data, such as BRAMS scores, is not advised [51]. For the assessment of multivariate normality, Mahalanobis distances (*p* < 0.001) identified 37 potential multivariate outliers. Each was systematically reviewed for indicators of response bias, including acquiescence, extremity, and straight-line answering patterns [50,52]. No evidence of such biases was detected. Consequently, no cases were removed, and the full sample of 417 BRAMS responses was included in the analyses.

To enhance methodological rigour and analytical comprehensiveness, multiple complementary analyses were conducted: (1) exploratory analysis, involving descriptive statistics and graphical visualisation of variable relationships [53]; (2) logistic regression with ElasticNet regularisation for injury prediction [47]; (3) survival analysis using Kaplan–Meier curves and Cox proportional hazards models to examine injury risk over time [54]; (4) random forest modelling to evaluate predictive accuracy and variable important [48]; (5) Bayesian estimation of injury probabilities and odds ratios between extreme mood profiles [49]; (6) bootstrap procedures for robust estimation of confidence intervals [55]; (7) statistical power analysis to evaluate the adequacy of the sample size in detecting medium-sized effects [51].

### 3.2. Mood and Injury Occurrence

The overall prevalence of athletes reporting at least one injury during the period from April to July 2023 was 14.6% (61 athletes). Of those injured athletes, 21 athletes completed the BRAMS prior to sustaining their injury, 20 athletes completed it after injury, and the exact timing of injury relative to BRUMS completion was uncertain for the remaining 20 athletes. MANOVA showed significant mood differences, with small effect sizes, for depression, anger, and fatigue between athletes who had experienced at least *one* injury compared with those who had not been injured (Table 2). Injury was associated with higher scores for depression, anger, and fatigue, explaining 3.7% of the total variance in mood scores. Discriminant function analysis showed that athletes could be correctly classified into injury categories (injury vs. no injury) based on mood scores, with an overall classification accuracy of 68%.

While statistically significant, the effect sizes for mood differences between injured and non-injured athletes were relatively small, with depression, anger, and fatigue explaining 1.0%, 1.5%, and 1.6% of the variance, respectively. This suggests that while mood states contribute to injury risk, they represent just one of many factors in a complex injury causation model. Notably, fatigue emerged as the mood dimension with the strongest association with injury occurrence, which is particularly relevant given that some profiles, such as the shark fin profile, are characterised by elevated fatigue without corresponding elevations in other negative mood dimensions.

Table 3 presents the prevalence of injury across mood profile clusters, highlighting meaningful differences in injury risk among athletes with distinct affective patterns. Athletes classified under the iceberg profile, characterised by high vigour and low negative mood, exhibited the lowest injury prevalence (12.9%, *n* = 11) and the highest proportion of uninjured individuals (87.1%, *n* = 74), thereby serving as the reference group.

In contrast, the inverse Everest profile, associated with extremely high negative mood states and low vigour, displayed the highest injury prevalence (30.0%, *n* = 6) and an odds ratio of 2.88 (95% CI: 1.01–8.23), suggesting that these athletes were nearly three times more likely to experience injury than those reporting an iceberg profile. Nevertheless, this estimate warrants cautious interpretation given the relatively small sample size in this subgroup (*n* = 20).

The inverse iceberg profile, marked by elevated negative mood and reduced vigour, showed a similar injury rate (12.2%, *n* = 5) to the iceberg group, with an odds ratio of 0.93 (95% CI: 0.32–2.70), indicating no appreciable difference in injury risk. Athletes with the shark fin profile, characterised by very high fatigue and below-average scores on other mood dimensions, had a moderately higher injury rate (19.2%, *n* = 15), with an odds ratio of 1.60 (95% CI: 0.71–3.64).

The submerged profile, defined by below-average scores across all mood states, demonstrated a slightly lower injury rate (10.2%, *n* = 12) than the iceberg group, with an odds ratio of 0.76 (95% CI: 0.35–1.63), suggesting a potential but inconclusive protective effect. Lastly, the surface profile, typified by approximately average scores on all mood dimensions, showed an injury prevalence of 16.0% (*n* = 12), with an odds ratio of 1.28 (95% CI: 0.56–2.89), indicating a modest and statistically uncertain increase in injury risk.

These findings underscore the importance of interpreting odds ratios alongside their confidence intervals to assess the precision and practical relevance of observed differences. While some profiles show elevated or reduced risk compared to the iceberg baseline, wide confidence intervals reflect variability and suggest the need for larger samples to confirm these associations.

Table 4 shows the distribution of injury types by sex. Acute injuries constituted most injuries for both females (65.5%) and males (62.5%), with a total of 39 cases. Overuse injuries accounted for 34.5% of female injuries and 37.5% of male injuries, with 22 cases reported. Chi-squared analysis showed no significant relationship between injury type and athlete sex (χ^2^ = 0.06, *p* = 0.81).

A MANOVA compared mood scores at pre-injury and post-injury timepoints (Table 5). The multivariate statistic, although not statistically significant, indicated that the time of mood assessment (pre-injury or post-injury) accounted for 18.4% of the variance in mood scores, which may be of practical importance. Univariate analyses showed a minimal difference in tension scores between timepoints (η^2^ = 0.001). In contrast, depression scores were significantly higher post-injury than pre-injury, with a moderate effect size accounting for 11.3% of variance, suggesting the psychological effects of injury may include elevated depressed mood.

Anger scores were higher post-injury than pre-injury, although not significantly so, with a moderate effect size accounting for 6.2% of variance. Vigour scores were lower post-injury compared to pre-injury, with a moderate but non-significant effect size accounting for 7.0% of variance. Fatigue scores were higher post-injury than pre-injury with a moderate effect size explaining 8.6% of variance, and a *p*-value approaching significance. Confusion scores showed little variation, with a very small effect size explaining only 0.1% of variance. This suggests that the cognitive or mental clarity of the athletes remains largely unaffected by injuries.

### 3.3. Mood and CMJ Performance

Table 6 includes the results of a MANOVA to compare mood scores across two groups of athletes, grouped according to their CMJ performance. The sample comprises a total of 225 athletes, 146 of whom improved their CMJ performance from the first to the second testing period (improved performance group) and 79 whose CMJ performance declined from the first to the second testing period (reduced performance group). The results showed a minimal difference in the overall mood profiles between the improved and reduced performance groups, with a non-significant multivariate statistic that accounted for only 2.6% of the variance in mood scores. Examining individual mood dimensions, none of the univariate comparisons between improved and reduced performance groups were statistically significant, none explained even 1% of variance, and all effect sizes were in the negligible-to-small range. Fatigue scores came closest to showing a meaningful difference between groups, although even that effect size was small. In summary, mood scores were not associated with CMJ performance variation.

The minimal relationship between mood states and CMJ performance (global η^2^ = 0.026) contrasts with the more substantial relationship between mood states and injury occurrence. This suggests mood states may influence injury risk through mechanisms distinct from those affecting immediate physical performance. The complete absence of significant univariate effects, coupled with negligible effect sizes across all mood dimensions (η^2^ between 0.000 and 0.009), provides robust evidence that acute power output as measured by CMJ is not meaningfully related to retrospective mood states in this athletic population.

This challenges simplistic assumptions about mood-performance relationships and suggests that pathways through which mood states influence athletic outcomes may be more complex than direct effects on power output. Mood disturbances may impact injury risk through indirect mechanisms such as attentional focus, decision-making, or risk-taking behaviour rather than immediate neuromuscular performance. Additionally, explosive, short-duration tasks like CMJ may be less susceptible to mood influences than tasks requiring sustained concentration, endurance, or complex cognitive processing.

### 3.4. Mood Profiles and Injury Risk: Key Patterns and Predictive Insights

Mood profile distributions mirrored patterns reported by Terry and Parsons-Smith [7], with the shark fin (28.3%) and iceberg (20.4%) profiles emerging as the most prevalent profiles within the sample, consistent with a previous large-scale BRUMS dataset [7]. Notably, the inverse Everest profile, which has been consistently associated with psychological vulnerability, demonstrated the highest injury rate (30.0%), aligning with prior findings that this profile reflects an extreme maladaptive mood state [7].

Although negative mood states (e.g., tension, depression, anger, fatigue, confusion) exhibited strong intercorrelations—as is typical in mood profiling literature—their individual associations with injury and performance outcomes were weak. This echoes the observations of Terry and Parsons-Smith [7], who caution that while mood assessments are valuable, their predictive validity is maximised when interpreted within a broader biopsychosocial context.

In line with these insights, logistic regression yielded limited predictive power (AUC = 0.502), identifying anger, vigour, and fatigue as marginal predictors of injury. Conversely, random forest modelling offered improved discrimination (AUC = 0.651), reinforcing the notion that non-linear, data-driven approaches may better capture the complexity of mood–injury dynamics. Survival analysis further supported the elevated injury risk for inverse Everest athletes, who were significantly more likely to be injured compared to those with shark fin (*p* = 0.0081) and iceberg (*p* = 0.0362) profiles.

Bayesian estimation and bootstrap procedures confirmed an increased likelihood of injury for athletes exhibiting inverse Everest profiles (OR ≈ 3.0), consistent with Terry and Parsons-Smith’s conceptualisation of this profile as acutely maladaptive [7]. However, statistical power analyses revealed that the current sample was underpowered to detect medium-sized effects, suggesting that the generalisability of predictive models remains limited without larger-scale validation.

## 4. Discussion

### 4.1. Mood Profiles as Indicators of Injury Risk

The differing injury rates associated with various mood profiles provide compelling evidence for the role of affective states in modulating physical vulnerability. Athletes exhibiting the highly negative inverse Everest profile were found to be at substantially greater risk of injury (OR = 2.88) compared to those reporting the iceberg profile. This supports foundational models such as the Stress and Injury Model proposed by Andersen and Williams [56], which posits that heightened emotional stress can impair focus, coordination, and physiological preparedness, thereby increasing susceptibility to injury. Elevated levels of depression, anger, and fatigue may compromise psychophysiological resilience, diminishing attentional control and increasing the likelihood of injury.

Conversely, the apparent protective association of the iceberg profile—characterised by high vigour and low levels of negative affect—reinforces the link between positive mood states and enhanced resilience. This is consistent with Lane et al. [57], who suggest that positive emotional states broaden cognitive and behavioural repertoires, improving problem-solving and adaptability in the face of physical and psychological stress. Athletes with elevated vigour may therefore possess superior adaptive resources, allowing them to buffer the effects of high training loads or competitive pressures. These findings support the incorporation of routine mood profiling as a preventive strategy, enabling practitioners to detect early signs of psychological strain and implement targeted interventions to mitigate injury risk. Importantly, negative mood states rarely act in isolation. Their combination with factors such as excessive training load, inadequate recovery, or prior injury likely results in a cumulative effect that magnifies injury vulnerability. This interaction supports a more integrated approach to athlete monitoring—one that concurrently tracks psychological and physiological indicators of readiness.

Fredrickson [58] introduced the Broaden-and-Build Theory of Positive Emotions, a foundational framework within positive psychology that explains how emotions such as joy, interest, and contentment broaden individuals’ momentary thought–action repertoires and, over time, help to build enduring personal resources. Unlike negative emotions, which tend to narrow cognitive and behavioural responses to immediate threats, positive emotions expand awareness and encourage novel, exploratory, and socially integrative behaviours. These broadened responses, in turn, facilitate the development of psychological, social, and physical resources that enhance well-being and resilience. Fredrickson’s work provides a theoretical basis for understanding the long-term adaptive value of positive emotional experiences in both health and performance contexts. Managing emotional states, therefore, becomes critical in maintaining optimal cognitive performance in high-pressure sports environments in which mood disturbances are more likely to affect tasks requiring sustained attention and executive function than brief, high-intensity efforts. Stange [59] further expanded on this integrated perspective, proposing that dynamic psychophysiological markers—such as heart rate variability and electrodermal activity—can serve as real-time indicators of risk and resilience. By monitoring these indices across time and situational contexts, Stange outlined a framework for identifying modifiable mechanisms underlying both vulnerability and adaptation, offering a personalised path to injury prevention and performance optimisation.

### 4.2. Stability of Performance Metrics in Relation to Mood Variability

The present study findings indicate a minimal connection between mood states and acute power-based performance, as measured by the CMJ. The non-significant results for mood effects on CMJ performance underscore that, while mood disturbances correlate with injury risk, they may not directly impact immediate power outputs in specific neuromuscular tasks. This observation is consistent with previous research suggesting that mood states exert a more pronounced effect on sustained or cognitively demanding tasks rather than on discrete power assessments. For instance, a meta-analysis by Lane et al. [57] found that mood states had a more substantial impact on endurance and cognitively demanding tasks compared to short-duration power tasks. Similarly, studies have shown that negative mood states impair executive functions such as problem-solving and cognitive flexibility, which are essential for decision-making and performance under pressure. In a sporting context, this suggests that athletes experiencing negative moods may struggle with tactical adaptability, concentration, and strategic execution during competition.

The stability of CMJ performance despite mood variations may reflect the primarily mechanical and neuromuscular nature of this specific test, which relies less on psychological factors than physiological capabilities. Unlike endurance or technically complex tasks where psychological factors might play larger roles, explosive power production may be more resistant to mood influences. The non-significant mood–performance relationship observed also highlights the multidimensional nature of athletic performance, where psychological, physiological, and environmental factors converge. While mood may not acutely impact power-based measures such as CMJ, prolonged exposure to mood disturbances could accumulate to affect motivation, fatigue management, and adaptability—elements critical to long-term athletic development and consistency in competitive performance. This underscores the necessity for holistic athlete monitoring approaches that integrate psychological and physiological metrics to create a comprehensive profile for each athlete.

### 4.3. Practical Implications: Integrating Mood Profiling into Athlete Monitoring Systems

The practical contribution of the study findings lies in its demonstration of mood profiling as a predictive tool for injury prevention and as an adjunct to physical performance evaluations. By incorporating BRAMS assessments into the multidisciplinary team protocol, this study demonstrates the value of an interdisciplinary approach to athlete monitoring that transcends traditional physical metrics to also include psychological indicators of well-being. This integration aligns with the vision of knowledge translation in sports science, promoting evidence-based interventions that bridge the gap between research insights and practical applications in the field [36]. The utility of BRAMS as a mood assessment tool allows for the identification of chronic mood disturbances, which may serve as early indicators of physical or psychological strain, thereby enabling timely interventions such as adjusted training loads, enhanced rest protocols, or psychological support.

The implications for practitioners are profound, as mood profiling not only identifies athletes at elevated risk of injury but also facilitates tailored interventions that consider both psychological readiness and physical capacity. This athlete-centric model resonates with contemporary sports science frameworks, which emphasise personalised training, recovery, and psychological support strategies that adapt to the unique needs of each athlete. By routinely assessing and responding to mood states, coaches and the health team can develop a more agile and responsive support structure that anticipates and mitigates risk factors before they culminate in injury or performance decrements.

Gabbett [60] advanced the “training–injury prevention paradox”, cautioning against abrupt increases in training load due to their association with elevated injury risk. Rather than simply reducing training volume to prevent injury, Gabbett advocated for smarter, progressive loading strategies that build athletes’ resilience and tolerance to workload. This approach emphasises the importance of structured monitoring and gradual progression to not only reduce injury incidence but also optimise performance and long-term athlete development.

### 4.4. Integrated Approaches to Athlete Monitoring and Injury Prevention

The findings suggest that while mood profiles significantly associate with injury risk, they explain a relatively small portion of total variance in injury occurrence (3.7%). This underscores the importance of adopting integrated, multifactorial approaches to athlete monitoring and injury prevention. Future research and practical applications should consider combining mood assessment with other monitoring strategies, such as training load quantification, sleep quality measurement, and physiological recovery markers. This multimodal approach would likely yield more powerful predictive models than any single factor in isolation. For example, combining elevated negative mood states with high acute:chronic workload ratios might identify athletes at particularly high risk for injury, enabling more targeted preventive interventions. Integrated psychophysiological indices can help identify risk markers, mechanisms, and intervention targets.

Williams and Andersen’s [61] seminal review critically examines the stress and injury model, which posits that psychosocial factors influence an athlete’s risk of injury primarily through stress responses. The model highlights three key antecedents: a history of stressors, personality characteristics (e.g., high competitive anxiety, low self-esteem), and limited coping resources. These elements interact to intensify physiological (e.g., muscle tension) and attentional (e.g., narrowed visual field) responses to stress, thereby increasing susceptibility to injury. The authors advocate for interventions targeting stress reduction and enhancement of coping mechanisms, such as relaxation training and social support. While the model has significantly advanced the understanding of sport injury aetiology, the authors call for further research into the dynamic interplay of risk factors and the contextual influences of different sporting environments, along with the development of individually tailored injury prevention programmes.

**Figure 2:**
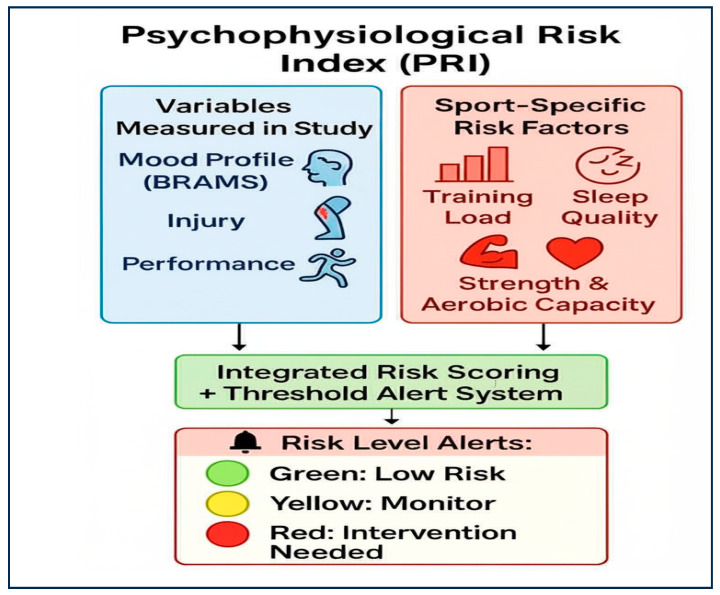
Proposed Psychophysiological Risk Index (PRI) Integrating Study-Measured Variables and Sport-Specific Risk Factors. Note: The model requires validation to test its efficacy in reducing the incidence of injury.

Figure 2 illustrates a proposed Psychophysiological Risk Index (PRI) that integrates multiple factors known to influence injury risk. This model combines assessment of mood profiles (via BRAMS), injury history, with sports specific factors training load metrics (such as acute:chronic workload ratio), sleep quality measures, and physical qualities such as strength and aerobic capacity into a composite risk score. The resulting integrated assessment provides a more comprehensive evaluation of athlete risk status, enabling more effective and precise interventions. The colour-coded alert system (green for low risk, yellow for monitoring required, and red for intervention needed) provides a clear, actionable framework for coaches and support staff. The findings suggest that while mood profiles significantly associate with injury risk, they explain a relatively small portion of total variance in injury occurrence (3.7%). This underscores the importance of adopting integrated, multifactorial approaches to athlete monitoring and injury prevention [61].

Future research and practical applications should consider combining mood assessment with other monitoring strategies, such as training load quantification, sleep quality measurement, and physiological recovery markers and intervention targets in real time. This perspective aligns closely with Andersen and Williams’ predictive and preventive stress-injury model [56], as well as the findings of Brenner and Watson [62], who highlight the crucial importance of monitoring both psychological strain and imbalances in training and recovery. These factors are key contributors to overuse injuries, burnout, and performance decline in athletes. By adopting a multi-dimensional, data-informed framework, coaches, sport scientists, and medical teams can implement a more proactive and individualised approach to athlete management—enhancing performance outcomes while safeguarding health and well-being, athlete management, and mitigating potential detriments to health and performance.

The development of individualised monitoring approaches also warrants consideration. Rather than relying solely on normative comparisons, tracking individual deviations from personal baseline mood states, as recommended by Terry [1], might provide more sensitive indicators of psychological strain. This individual-centred approach acknowledges the substantial variability in how athletes respond psychologically to similar training stimuli and competitive pressures. The implementation of such integrated monitoring systems requires careful consideration of practical constraints. Digital platforms that facilitate regular mood assessments with minimal burden on athletes, combined with automated flagging of concerning patterns, could enhance compliance and timely intervention.

### 4.5. Future Directions and Methodological Considerations

Several methodological considerations warrant discussion when interpreting the current findings. First, our research design precludes causal inferences regarding the relationship between mood states and injury occurrence. Although our results demonstrate associations between negative mood profiles and injury risk, it was shown among injured athletes that post-injury mood was more negative than pre-injury mood, suggesting that the negative impact of injury on mood is stronger than the impact of pre-existing negative mood on risk of injury. Longitudinal research with repeated mood assessments over an extended period would provide stronger evidence regarding the directionality of these relationships.

Second, sample size limitations affected certain analyses, particularly for less common mood profiles and the pre/post-injury comparison. The inverse Everest profile, while showing the strongest association with injury risk (OR = 2.88), included only 20 athletes (6 injured), resulting in relatively wide confidence intervals around this estimate. Similarly, the pre/post-injury comparison (*n* = 41) was underpowered for detecting small-to-moderate effects, potentially explaining why the substantial multivariate effect size (η^2^ = 0.184) did not reach statistical significance.

Third, our binary categorization of continuous variables, such as CMJ performance (improved vs. reduced), represents a simplification that may have obscured more nuanced relationships. Future research might benefit from analysing raw performance changes or establishing clinically meaningful thresholds for performance improvement.

Despite these limitations, the consistent pattern of results across analyses lends credibility to our primary finding that negative mood states, particularly depression, anger, and fatigue, are associated with increased injury risk in athletes. The contrast between the significant mood–injury relationship and the non-significant mood–performance relationship is particularly noteworthy and suggests different pathways through which psychological factors influence athletic outcomes.

These findings point to the potential benefits of hybrid mood assessment protocols combining both retrospective and immediate response frames. Such an approach could capture short-term mood fluctuations in addition to chronic mood patterns, offering a more nuanced perspective on the relationship between mood, injury, and performance. A hybrid mood monitoring approach combining retrospective (“past week”) and immediate (“right now”) mood states could offer richer temporal resolution and early detection of emerging risk factors, enhancing responsiveness of preventive strategies. Longitudinal research with diverse populations is recommended to further delineate cultural and environmental influences on mood-performance dynamics, as well as the implications of mood profile shifts over time.

## 5. Conclusions

This study underscores the critical role of mood profiling as an integral component of athlete health monitoring, particularly for identifying injury risk in elite sports settings. The findings highlight the value of specific mood profiles, such as the inverse Everest, in recognising athletes at heightened injury risk, while also emphasising the protective qualities associated with the iceberg profile. These insights support a preventative, proactive approach to athlete care, wherein psychological assessments are used alongside physical performance metrics to inform training, recovery, and injury prevention strategies.

The implications of the present study are particularly relevant to sports practitioners seeking to employ a holistic approach to athlete management. By integrating mood assessments into routine performance evaluations, practitioners can apply a multidimensional framework that identifies potential injury risks, optimises training conditions, and supports athletes’ psychological well-being. This model not only aligns with contemporary sports science principles but also exemplifies a progressive application of knowledge translation in sport, bridging the gap between psychological research and practical, actionable strategies in athlete care.

Our findings regarding the relationship between mood profiles and injury risk, while statistically significant, should be interpreted within the context of the relatively small effect sizes observed (explaining 1.0–1.6% of variance). This suggests that mood states represent one component within a multifactorial injury risk model rather than a dominant predictive factor. Nevertheless, the practical significance of identifying psychological risk factors should not be underestimated, as mood states are modifiable through appropriate interventions, potentially offering a cost-effective approach to injury prevention that complements physical and technical training adjustments.

The non-significant relationship between mood and immediate power performance (CMJ) juxtaposed against the significant mood-injury relationship represents an important contribution to our understanding of how psychological factors impact athletic outcomes. This pattern suggests that the influence of mood on injury risk is likely to operate through mechanisms other than direct impairment of neuromuscular power, such as attentional processes, decision-making, or recovery behaviours. Future research employing longitudinal designs with mediation analyses could further elucidate these pathways.

Integrating psychological monitoring with physical and training load assessments holds promise for developing more comprehensive athlete management systems. Such integrated approaches would enable the identification of complex risk patterns—such as the combination of negative mood states with high training loads or inadequate recovery—that may predispose athletes to injury more strongly than any single factor in isolation.

Although mood profiles alone offer limited predictive power, their value becomes significantly enhanced when interpreted alongside objective measures such as training load and recovery status. It is through this integrated, multidimensional approach that practitioners can more accurately identify athletes at heightened risk of injury and apply timely, targeted interventions. The convergence of psychological and physiological monitoring represents a meaningful advancement in personalised athlete care—one that not only deepens our understanding of injury vulnerability but also optimises long-term performance and well-being. This synthesis of affective and physical data should therefore be positioned not as supplementary, but as central to modern athlete management frameworks.

## Figures and Tables

**Table 1:** Demographic and sport distribution of the sample (*n* = 417).

Source	Group	*n*	*%*
Sex	Male	229	54.9
	Female	188	45.1
Age Group	12–17 years	252	60.4
	18+ years	165	39.6
Sport	Artistic Swimming	24	5.8
	Basketball	22	5.3
	Gymnastics	10	2.4
	Judo	35	8.4
	Rowing	98	23.5
	Swimming	70	16.8
	Volleyball	83	19.9
	Water Polo	75	18.0
Total		417	100.0

**Table 2:** Results of a MANOVA to compare mood scores by injury occurrence (*n* = 417).

Source	No Injury (*n* = 356)		Injury (*n* = 61)		*F*	*p*	*d*	*η* ^2^
	*M*	*SD*	*M*	*SD*				
Tension	45.88	8.07	47.54	9.71	2.06	0.152	0.20	0.005
Depression	51.99	12.37	55.69	18.09	3.99	0.047 *	0.28	0.010
Anger	53.59	15.07	59.30	22.76	6.31	0.012 *	0.35	0.015
Vigour	45.91	8.52	45.89	8.45	0.01	0.985	0.00	0.000
Fatigue	56.68	12.51	61.20	13.62	6.61	0.011 *	0.35	0.016
Confusion	49.33	9.36	49.41	11.55	0.00	0.955	0.01	0.000

Note: Wilks lambda = 0.963, *F*(6,410) = 2.65, *p* = 0.016, partial eta-squared = 0.037. * *p* < 0.05.

**Table 3:** Prevalence of injury by mood profile cluster.

Profile	No Injury (*n* = 356)		Injury (*n* = 61)		Total	%	Odds Ratio vs. Iceberg
	n	%	n	%			
Iceberg	74	87.1	11	12.9	85	20.4	-
Inverse Everest	14	70.0	6	30.0	20	4.8	2.88
Inverse Iceberg	36	87.8	5	12.2	41	9.8	0.93
Shark Fin	63	80.8	15	19.2	78	18.7	1.60
Submerged	106	89.8	12	10.2	118	28.3	0.76
Surface	63	84.0	12	16.0	75	18.0	1.28

**Table 4:** Type of injuries sustained grouped by athlete sex.

Injury Type	Female Injury (*n* = 29)		Male Injury (*n* = 32)		Total
	*n*	%	*n*	%	
Acute	19	65.5	20	62.5	39
Overuse	10	34.5	12	37.5	22

**Table 5:** MANOVA to compare mood scores pre- and post-injury (*n* = 41).

Source	Pre-Injury (*n* = 21)		Post-Injury (*n* = 20)		*F*	*p*	*d*	*η* ^2^
	M	SD	M	SD				
Tension	47.71	9.69	47.05	9.78	0.05	0.828	0.07	0.001
Depression	49.00	7.12	57.40	17.09	4.30	0.045 *	0.62	0.113
Anger	52.33	12.56	59.60	18.18	2.24	0.143	0.46	0.062
Vigour	47.14	9.45	42.70	7.73	2.70	0.108	0.50	0.070
Fatigue	57.52	13.85	65.50	11.71	3.95	0.054	0.60	0.086
Confusion	46.67	5.99	47.70	8.34	0.21	0.650	0.14	0.001

Note: Wilks lambda = 0.816, *F*(6,34) = 1.28, *p* = 0.292, partial eta-squared = 0.184. Twenty injured athletes were excluded from this analysis because the precise date of injury was uncertain.

**Table 6:** MANOVA of mood scores by CMJ performance (*n* = 225).

Source	Reduced Performance (*n* = 79)		Improved Performance (*n* = 146)		*F*	*p*	*d*	*η* ^2^
	M	SD	M	SD				
Tension	45.10	8.47	46.29	8.32	1.04	0.309	0.14	0.005
Depression	51.42	9.96	51.49	10.49	0.00	0.962	0.01	0.000
Anger	52.84	12.30	54.71	17.30	0.73	0.394	0.11	0.003
Vigour	46.14	8.90	46.47	8.40	0.08	0.781	0.04	0.000
Fatigue	60.33	13.01	57.82	13.02	1.90	0.169	0.19	0.009
Confusion	48.03	8.62	48.60	7.72	0.26	0.612	0.08	0.001

Note: Wilks lambda = 0.974, *F*(6,218) = 0.97, *p* = 0.449, partial eta-squared = 0.026.

## Data Availability

Restrictions apply to the availability of these data. Data were obtained from Clube de Regatas do Flamengo, Rio de Janeiro, Brazil and are available from the corresponding author with the permission of Clube de Regatas do Flamengo.
